# Progress in Medicine

**Published:** 1899-01-07

**Authors:** 


### Progress in Medicine.

### DIABETES.

Fitz and Joslin[1] give a summary of the cases recorded in the Massachusetts General Hospital, from 1824 to the present time, numbering 172, among whom only one negro appeared. Chemical tests for sugar were only employed since 1851; eighteen out of 47 patients died comatose; disease of the pancreas was only noted once. Tyson thinks that the disease is more common among negroes than is supposed, though they have a comparative immunity. N. S. Davis, in relating the history of some cases of gangrene, speaks of the occasional disappearance of sugar during the process, and of a similar change occurring when tuberculous disease is active in the lungs. He advises operative interference when gangrene has attacked the toes so soon as a line of demarcation has been formed, a view which has been opposed by former writers, from the fear of septic complications.

**Primary Cancer of the Pancreas** has been recorded about 150 times, but Bard and Pic[2] find that only in 17 of these was glycosuria observed. It is possible that it existed, however, in many more, and on the other hand a deduction would have to be made for those cases where the glycosuria was the primary affection, and the cancer a result of it. The writers notice, too, that the glycosuria may appear in the earlier stages of the disease from the sclerosis produced by the pressure of the tumour on the duct, while its frequent disappearance in the later stages is due, they think, to the cancer cells taking on the physiological functions of those of the pancreas. This, however, is opposed to the analogy of other cancerous growths. The symptoms of the disease are emaciation, cachexia, bronzing, distension of the gall bladder, and sometimes pain and a tumour, together with the absence of ascites and of any swelling of the liver and spleen. These may be accompanied by either simple glycosuria or by the complete symptoms of diabetes, or by lipuria or steatorrhœa. Polyakoff thinks that pancreatic calculus may be marked by colic with vomiting, followed by persistent pain and wasting, and sometimes temporary diabetes. He describes a case in support of this view.

The controversy between Noel Paton[4] and Pavy on the pathology of diabetes is still going on. The former holds that the liver stores up any excess of carbohydrates in the form of glycogen, and when sugar is needed forms it from this store and from other substances and passes it into the blood to be used up in the tissues. Pavy denies that the glycogen is converted into sugar, and holds that any sugar in the blood is thrown off by the kidneys. In defence of his view, Paton refers to the easy convertibility of glycogen into sugar, its decrease and the increase of glucose in the liver when the circulation is stopped, or when the floor of the fourth ventricle is punctured. In other abnormal conditions, too, glycogen is thus converted. Pavy would argue that this increased production in special cases is due to the formation of a special zymin in the liver after death, but Paton shows experimentally that such a zymin is not formed post-mortem, and points out that the sugar in the hepatic vein often exceeds that in the portal, and even in starving animals is greater than that in the arteries. Besides this, the sugar in the blood is shown by recent observers to be reduced when the liver is shut off from the circulation, while Harley and Voit prove that injections of certain sugars into the blood are not wholly excreted by the kidneys, but are actually used up in the tissues as shown by the increased respiratory quotient. Even large quantities are thus absorbed in human beings without harm. Pavy[5] finds that the proof of the excess of sugar in the hepatic vein is fallacious, and though he argues that the experiments on the retention of injected sugar within the organism are contradictory, he holds that the entire organism does possess the power of changing sugar into glycogen.

Marcuse[6] shows that phloridzin does not increase the sugar in the blood, but forms it only at the moment of excretion in the kidney. Thus methylene blue given to a patient who took also phloridzin still produced blue urine, while if he ate a large quantity of grape sugar the urine was decolorized by the sugar in the blood. If it is true that the sugar in diabetes is lessened by methylene blue, that result is obtained by the oxidation which the methylene exerts on the sugar in the blood. The formation of sugar by phloridzin appears, moreover, to take place in the tubules, and not in the glomeruli of the kidney. Bouchard[7] regards diabetes as caused by a diminution of the destruction of sugar by the tissues, and not by an increased production. He remarks that if we find 3 oz. of sugar in the urine on a strict albuminous diet, and if the normal destruction of sugar in the organism is kept up of 17 oz. daily, we should need for its supply about 10 pounds of albuminous food or the destruction of an equal quantity of the body tissue daily—a perfectly impossible state of things. The essence of the disease then is a reduced consumption of sugar by the tissues, a failure of their capacity for nutrition.

**Albuminuria in Diabetes.**—L. Goudard[8] distinguishes two forms—functional and pathological. The former may, if neglected, pass into true nephritis, but is primarily due either to fatigue of the kidney, phosphaturia, hyperchlorhydria, or organic disassimilation. The true nephritis is shown by the presence of large quantities of albumen and of cylindrical epithelium, the alkalinity of the urine, and cardiac changes. Diabetic diet should be rigidly adhered to in the functional form, but in the severe form we should use that adopted for Bright's disease, and even give an exclusive milk diet when the kidney lesion requires it. Some statistics are given on the same subject by Karl Grube,[9] who found albumen in 191 cases out of 473 of diabetes, and quotes other authorities as recording it in from 23 to 67 per cent. He divides the instances into (*a*) albuminuria occurring in the last stage of diabetes and in coma produced by a hyaline or glycogenic change in the epithelium of Henle's loops and the straight tubules; (*b*) that arising from heart failure; (*c*) senile albuminuria; (*d*) the functional form arising from the irritation of sugar and disappearing with it; (*e*) that arising in true Bright's disease to which diabetes sometimes gives rise. The kind of Bright's disease produced is nearly always that of parenchymatous nephritis, and it usually follows a severe form of diabetes. It must be carefully distinguished from granular kidney, which is not infrequently accompanied by glycosuria of a mild type. He advises milk when well tolerated, warm baths, and absolute rest.

**Tests.**—Bremer[10] finds that if a drop or two of gentian violet finely rubbed up be poured on the surface of normal urine no colour is produced, but if the same test be applied to diabetic urine the upper layers are coloured blue or violet, and the colour does not disappear on shaking. With urines of a specific gravity below 1015, and in cold weather the test may fail, and in some cases where the metabolism of the body is defective a slight colour may be seen in otherwise normal urine. The test may be of great value as a preliminary one in saving the boiling process on which we usually rely. The action seems to be due to some reducing substance present in the diabetic urine and not to the sugar itself. It is best to use Merck's "Gentian violet B." Loewy[11] has simplified Bremer's blood test. He stains a film for two minutes in a 2 per cent. solution of methylene blue, and then for ten seconds in a 0·125 per cent. eosin solution. If diabetes is present with more than 2 per cent. of sugar in the urine, the corpuscles are yellow, but in health they are stained a deep brown. It may be mentioned that Ebstein has shown that the diabetic excretes less carbon dioxide by the lungs than a healthy person under similar conditions.[12] It is not clear what are the substances which are active in the above tests for diabetes. Saundby[13] quotes Kolisch as finding a quantity of jecorin in the blood of these patients, a substance which can be decomposed into sugar and lecithin, and there are possibly certain ferments also. Bremer's own method consists in heating the smeared slides for six to nine minutes at a temperature of 135 deg., and then staining with a 1 per cent. solution of Congo red, methyl blue, or Biebrich scarlet for one and a half or two minutes. After this they are rapidly washed in water and dried. Diabetic blood is stained by the last, but not by the first two; normal blood is the reverse. The phenyl-hydrazin test, though very delicate, is not of practical value according to Holmgrew,[14] for any alkaline reaction interferes with its success, and, above all, it requires to be supplemented by determining the melting point of the crystals. In healthy persons crystals of pentose occur, which can only be distinguished by finding that their melting point differs from that of grape sugar—a difficult and tedious process. R. Benjamin[15] recommends Lehman's new iodine method of estimating sugar for its accuracy. A fixed quantity of Fehling's solution, say 60 cc., is boiled with 5 cc. of diabetic urine and passed through a fine filter. The filtrate and washings are made up to 250 cc. Then 50 cc. of this is treated with iodine of potash and acidulated with $H_2SO_4$. The iodine is finally measured by hyposulphite of sodium in the usual way. He finds that 315 parts of copper correspond to 165·3 parts of sugar.

**Treatment.**—Leo, of Bohn,[16] employs a rigid diet for three to five weeks, and afterwards gives a small amount of carbohydrates, recurring to the rigid diet three times a year in severe cases. Von Jaksch[17] had found some success in giving arabinose, a sugar derivative, which, like lævulose, is easily assimilated by the diseased organism. Lemoine,[18] when he fears the onset of coma, gives ten grains each of lithium carbonate and antipyrin in a glass of alkaline water thrice a day. The antipyrin is continued for a fortnight, and increased to even three grammes a day, and the lithia is raised to two grammes. They are, however, diminished as the symptoms improve, and after the fortnight it is often possible to cease the antipyrin for a week. A second course of fourteen days usually causes the disappearance of all sugar. The treatment is applicable only to gouty glycosuria. The various causation of diabetes is shown by two cases successfully treated by Pietro Lupo with a purely vegetable diet, without alcohol,[19] but including ripe fruit and grapes.

The Editor of the *British Medical Journal*[20] contrasts some brands of gluten bread, containing 30 to 40 per cent. of carbohydrate, with the 15 per cent. of potatoes and the 16 per cent. of artichokes. Apples contain only 11·7 per cent., and this is reduced by cooking to nearly half. If the water in which fruits are stewed is thrown away and replaced by a fresh supply, with saccharin, nearly all the sugar is got rid of. No better effect is obtained by giving nine ounces of gluten bread, with 38 per cent. starch, than is produced by six ounces of white bread, with 60 per cent. Toast also is no better than bread, while milk can be taken in large quantities if passed through a separator to remove the sugar [and then thickened with egg].

James Oliver[21] finds that cocaine, in doses of a third of a grain two or three times a day, is helpful in overcoming the constipation of diabetes, and in the same paper records a case of coma which recovered after an injection of two and a half pints of saline solution into the veins, and free purgation. No relapse took place during the next four weeks, which is an almost unique success.

[1] Med. Rec., June 18. [2] Amer. Jour. Med. Sci., April. [3] Brit. Med. Jour., April 16. [4] Brit. Med. Jour., Feb. 26. [5] Brit. Med. Jour., April 2. [6] La Semaine Med., April 16. [7] La Semaine Med., May 14. [8] Med. Rec., May 28. [9] Brit. Med. Jour., July 23. [10] Brit. Med. Jour., May 21. [11] Brit. Med. Jour., May 7. [12] Ibid. [13] Birmingham Med. Review, Aug. [14] Amer. Med. and Surg. Bull., Feb. 25. [15] Lancet, Sept. 17. [16] Lancet, May 21. [17] Med. Rec., May 7, and La Semaine Med., April 20. [18] Med. Rec., June 25. [19] Therapist, June 15. [20] Brit. Med. Jour., July 15. [21] Lancet, Aug. 13.

